# The Effect of Maternal High-Fat Diet on Adipose Tissue Histology and Lipid Metabolism-Related Genes Expression in Offspring Rats

**DOI:** 10.3390/nu16010150

**Published:** 2024-01-02

**Authors:** Sabriye Arslan, Hilal Yıldıran, Cemile Merve Seymen

**Affiliations:** 1 Department of Nutrition and Dietetics, Faculty of Health Sciences, Gazi University, Ankara 06490, Turkey; hilalciftci@gazi.edu.tr; 2 Department of Histology and Embryology, Faculty of Medicine, Gazi University, Ankara 06500, Turkey; cmerveseymen@gazi.edu.tr

**Keywords:** pregnancy, lactation, maternal nutrition, high-fat diet, adiposity, adipose tissue, FAS, PPAR-γ, SREBP-1c

## Abstract

The developing fetus is dependent on the maternal nutritional environment. This study was conducted to determine the effects of a maternal high-fat diet (HFD) applied during pregnancy and/or lactation on the expression levels of some lipid-related genes in rat models. Half of the pregnant rats (n: 6) were fed an HFD (energy from fat: 45%), while the other half (n: 6) were fed a control diet (CD) (energy from fat, 7.7%) during the pregnancy period. During lactation, dams in both groups were divided into two subgroups, with half fed the CD and the other half fed the HFD. Thus, four groups were obtained: CD-CD, CD-HFD, HFD-CD, and HFD-HFD. At the end of lactation, all mothers and half of the offspring were sacrificed. The remaining offspring were fed a CD for five weeks. The average birth weight of the CD group offspring was found to be lower than that of the HFD group (*p* < 0.05). The amount of adipose tissue was highest in CD-HFD (*p* < 0.05), while gene expression levels were similar between groups (*p* > 0.05), and the most degenerative histological changes were observed in the eight-week HFD-HFD (*p* < 0.05). This study suggests that maternal HFD during pregnancy and lactation may increase adiposity in offspring rats, especially during the weaning period.

## 1. Introduction

Obesity, which has approximately tripled worldwide since 1975, is recognized as a global epidemic [1]. Additionally, women of reproductive age are impacted by the rising prevalence of obesity. According to the results of the Turkey Demographic and Health Survey (TDHS) conducted in our country in 2013, 55.2% of women of reproductive age are overweight or obese [2]. According to the 2017 Turkey Nutrition and Health Survey (TNHS) data, 28.5% of women aged 19–64 are overweight, 33.1% are obese, and 6.2% are morbidly obese [3]. In this population, the increasing prevalence of obesity and its complications, especially during pregnancy and lactation, can negatively impact health by causing an imbalance in fetal nutrition during the developmental stages. Increasing obesity can lead to an increase in both maternal risks and risks of spontaneous abortion, fetal malformations, fetal macrosomia, stillbirth, and preterm birth [4]. The number of infants born to obese mothers has increased in parallel with the rising prevalence of obesity among women, subsequently leading to an increased risk of childhood overweight and obesity [5]. According to data from the Turkey Childhood Obesity Study-2016 (COSI-TUR-2016), 9.9% of second-grade elementary school students were found to be obese, and 14.6% were overweight [6]. When compared to the Turkey Childhood Obesity Study (2013) (COSI-2013) for children aged 7–8, it is observed that the number of obese and overweight individuals has increased [7]. When examining the World Health Organization’s 2022 Obesity Report, it is reported that Turkey has the highest prevalence of obesity in the European region [8].

The developing fetus is dependent on the nutritional milieu of the mother. Consequently, fetal cellular and organ development, gene expression, and the epigenome may be impacted by nutrient deficiencies or excesses, which may ultimately result in metabolic and functional changes. Barker et al. conducted preliminary research that established the correlation between insufficient nutrition and adult diseases, thereby introducing the theory of developmental programming [9]. According to the hypothesis ‘Fetal Programming’ or ‘Developmental Origins of Adult Health and Diseases’, suboptimal intrauterine conditions can lead to specific changes in the fetus’s developmental stages, potentially influencing the development of chronic diseases later in life. The initial studies reported that inadequate nutrition during pregnancy negatively affects fetal growth parameters [9]. However, metabolic developmental programming is not limited to inadequate nutrition; recent studies have shown that maternal excessive energy intake, maternal low protein intake, increased/reduced specific foods/nutrients (such as folate, B12 vitamin, choline, and other methyl donors, fish oil, *n*-3 fatty acids). A high-fat diet (HFD) also influences future offspring health [9,10,11]. 

The Western diet, characterized by higher fat consumption, is well recognized as a significant contributing factor to metabolic disorders, including obesity, type II diabetes, insulin resistance, dyslipidemia, and hypertension. In recent years, there has also been an increase in dietary fat intake in our country, as evidenced by the Turkey Nutrition and Health Survey (TNHS) in 2017, which identified an increase in both total fat and saturated fat intake compared to TNHS-2010 [3]. Approximately 35% of the daily energy intake of both pregnant and lactating women comes from fats [3]. Research conducted on rats has demonstrated that consuming a diet high in fat during pregnancy and lactation has a substantial effect on the body composition of offspring, increases the likelihood of metabolic syndrome, and contributes to the development of obesity in both early life and adulthood [12,13]. Furthermore, even mild maternal overnutrition induced by a high-fat diet has been demonstrated to lead to excessive fat accumulation, glucose intolerance, and alterations in brain appetite regulators in offspring [14].

Additionally, excessive nutrition and maternal adiposity can affect epigenetic mechanisms during adipogenesis. The aforementioned alterations may exhibit sustained effects on the expression of genes associated with adipogenesis and lipogenesis [15]. In general, the available evidence indicates that maternal overnutrition programs obesity through altered expression of key regulatory factors in adipose tissue [16]. Proliferator-activated receptors for lipids (PPARs) and sterol regulatory element-binding proteins (SREBPs) are two of the most important transcription factors recognized for their role in lipid metabolism gene expression regulation [17]. Fatty acid synthase (FAS) catalyzes the final stage of de novo synthesis of fatty acids [18].

The literature includes studies evaluating the effects of maternal diet during pregnancy or lactation, where offspring born to HFD-fed mothers are either breastfed by control diet (CD)-fed mothers or vice versa, a technique known as cross-fostering [12,19,20,21]. Such studies allow for a clear separation of prenatal and postnatal environmental exposures, but one limitation of this approach is that it may affect metabolic outcomes in offspring [19]. Therefore, in this study, modifying mothers’ diets during pregnancy and lactation has been preferred to assess the impact of maternal diets during pregnancy and lactation on offspring. This study is particularly significant for evaluating the effects of maternal HFD exposure during lactation and pregnancy without intervening in pre-pregnancy maternal and post-lactation conditions. In addition, previous studies have generally observed that the continuation of the mother’s diet type after weaning in offspring has made it challenging to understand the maternal diet’s effects. In this study, providing CD to the offspring after weaning may not only facilitate the examination of maternal diet effects but also help determine whether there is a change in programming in the offspring with the transition to the CD. This study investigated how a maternal HFD during pregnancy and/or lactation affects the adipose tissue morphology and lipogenic gene expression in offspring.

## 2. Materials and Methods

This research was approved by Gazi University’s Animal Experiments Local Ethics Committee as a research project with registration number G.Ü.ET-19.039 on 8 July 2019. The principles of the Gazi University Animal Experiments Local Ethics Committee carried out all stages of the study. The financial resource of the research was covered by the Gazi University Scientific Research Projects Unit (Project Code No: 47/2020-01). 

### 2.1. Study Design 

This study was conducted on Wistar-type rats at Gazi University Laboratory Animal Breeding and Experimental Research Center between August 2020 and January 2021. In this study, 12 Wistar-type female rats obtained from Gazi University’s Laboratory Animal Breeding and Experimental Research Center were included (mean body weight of the control diet (CD) group: 193.8 ± 5.23 g and high-fat diet (HFD) group: 198 ± 6.22 g, *p* > 0.05). Female rats, 8–10 weeks old and from the same strain that had not been mated previously, were included in the study. The animals were maintained in a controlled environment with the following conditions: room temperature: 21 ± 2 °C, relative humidity: 35–40%, cage light intensity: 40 lux, light period: 12 h light/12 h dark, noise level: below 85 dB.

**Figure 1:**
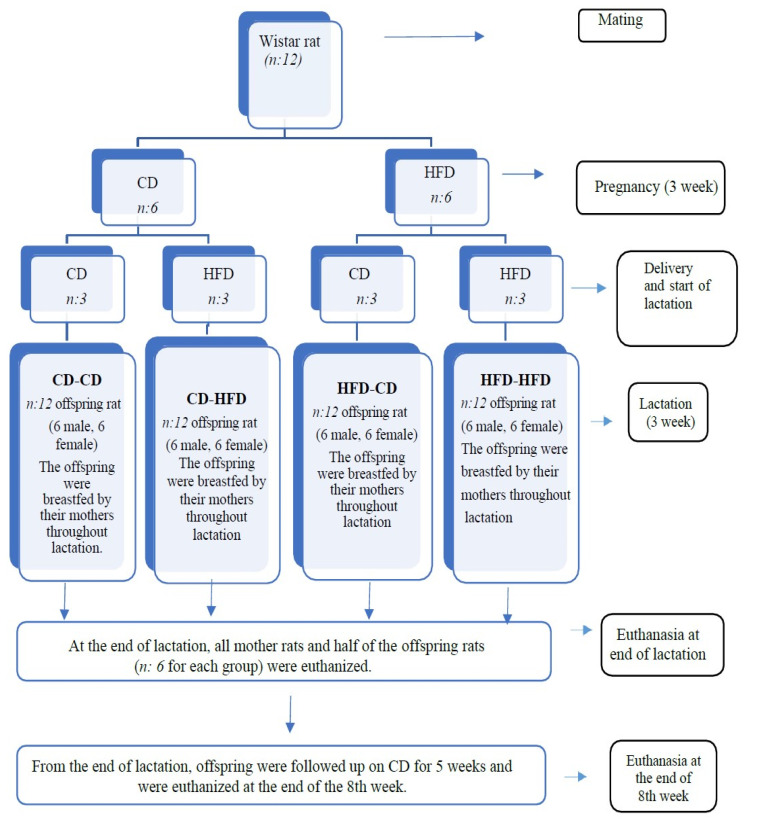
Study design.

Furthermore, the environment was equipped with a ventilation system capable of providing air exchange 10–16 times per hour. They were mated after the adaptation period, and the pregnant rats were randomly divided into control and experimental groups. Control group rats were fed a standard laboratory diet (Control diet: CD), and experimental group rats were fed a high-fat diet (HFD) throughout pregnancy. The experimental group received a diet with 45% of the energy coming from fat (a mixture of lard and soybean oil) (D12451, Research Diets). Both groups were provided ad libitum access to water and feed during pregnancy. During the lactation period, each group was divided into two groups again; half were fed with the CD (n: 3) and the other half were fed with the HFD (n: 3). Thus, the 1st group: with CD during pregnancy and lactation (CD-CD), the 2nd group: with CD during pregnancy and HFD during lactation (CD-HFD), the 3rd group: with HFD during pregnancy and CD during lactation (HFD-CD), 4th group: fed with HFD during pregnancy and lactation (HFD-HFD). At the end of the lactation period (3 weeks), dams and half of the offspring were sacrificed, and the remaining offspring were fed with CD until they were eight weeks old and were sacrificed at the end of the eighth week. The types of diets consumed by mother rats during pregnancy and lactation according to groups are shown in Table 1. During the offspring follow-up period after weaning, they were fed ad libitum with CD. A total of 60 rats, including 12 mothers and 48 offspring, were included in the study. Throughout pregnancy and lactation period, the mothers’ body weights and feed consumption were measured weekly at the same time (08.30–10.00) using a kitchen scale with a sensitivity of 0.1 g. The number of litters was recorded at the end of pregnancy. The birth weights of all offspring were measured with a precision scale. Until the end of the lactation period, the body weights of the offspring were measured every week. At the end of lactation, all mothers and half of the offspring (n: 24), as well as the offspring monitored for eight weeks postnatally (n: 24), were sacrificed by intracardiac blood collection under deep anesthesia after an average of 16 h fasting. A summary of the study design is shown in Figure 1.

### 2.2. Body Weight Measurements

Body weight gain in offspring was calculated using birth weight, body weight at the end of lactation, and body weight at the end of the study (final body weight). 

For the third week: Body Weight Gain (BWG) = Body weight at the end of lactation (g) − Birth weight (g).

For the eighth week: Body Weight Gain (BWG) = Final body weight (g) − Birth weight (g).

Offspring rat body weights and nose–anus length were measured before the sacrifice process. In evaluating the obesity of offspring rats, the Lee index was calculated. Rats with a Lee index greater than 0.3 were considered obese [22]. 

### 2.3. Blood Sample Collection and Evaluation of Biochemical Parameters 

The collected blood samples were centrifuged at 2500 rpm for 20 min using a Selecta-Centronic—BL model centrifuge. The separated serum samples were transferred to 1.5 mL Eppendorf tubes and stored at −80 °C until biochemical analysis was conducted. The researchers performed biochemical parameter analysis for rats at a specialized laboratory center. Serum insulin, glucose, triglyceride, total cholesterol, and leptin parameters in both mothers and offspring were evaluated using the quantitative Enzyme-Linked Immunosorbent Assay (ELISA) method with commercial kits. All analyses using ELISA kits were conducted following the manufacturer’s instructions. The Rat Glucose ELISA kit (Biossay Technology Laboratory, Shanghai, China), Rat Insulin ELISA kit (Biossay Technology Laboratory, Shanghai, China), Rat Leptin ELISA kit (Biossay Technology Laboratory, Shanghai, China), Rat Cholesterol ELISA kit (Biossay Technology Laboratory, Shanghai, China), and Rat Triglyceride ELISA kit (Biossay Technology Laboratory, Shanghai, China) were used for these biochemical analyses. All biochemical analyses were performed using appropriate methods.

### 2.4. Histological Evaluation of Adipose Tissue of Offspring

White adipose tissue (WAT) was obtained for histomorphometric analysis of adipose tissue. All tissue samples of adipocytes were initially fixed in 10% formaldehyde solution for light microscopic examination. Following fixation, tissue samples were placed in cassettes and washed under running water for 24 h. To remove water, tissues were passed through increasing degrees of alcohol (70%, 80%, 90%, and 100%). Afterward, the tissues were treated with xylene for polishing and then embedded in paraffin. Sections with a thickness of four microns obtained from prepared paraffin blocks were subjected to Hematoxylin–Eosin staining for all groups. Based on the evaluation using Hematoxylin–Eosin staining, a degeneration criteria table was created, taking into account the findings obtained as a result of the staining, and two independent researchers blindly examined all the preparations of all subjects and scored them. Numerical values for this table were established as follows: “0 = Absent, 1 = Mild, 2 = Moderate, and 3 = Severe”. The sections were evaluated in the LAS program on the Leica DCM 4000 (Germany) computer-aided imaging system, and images of the sections were captured. Histological analyses were conducted at the Department of Histology and Embryology, Gazi University Faculty of Medicine.

### 2.5. Evaluation of Gene Expression Analyses of Offspring

At a specialized company, offspring gene expression analyses were evaluated for FAS, PPAR-γ, and SREBP-1c genes in adipose tissue. Adipose tissue samples were collected in duplicate. The collected samples were transferred to Eppendorf tubes, frozen in liquid nitrogen, and appropriately transported to the laboratory in a cold chain by personnel responsible for sample transportation. Within the mRNA gene expression analysis scope, total RNA extractions were performed from 48 rat adipose tissue samples and real-time Polymerase Chain Reaction (PCR) analyses of FAS, PPAR-γ, and SREBP-1c genes. Beta-actin was used as a housekeeping gene for normalization in the experimental procedures. The process of obtaining gene expression data from the adipose tissue of offspring for FAS, PPAR-γ, and SREBP-1c genes involved the following steps:

Total RNA Extraction: TRIzol Reagent was used to ensure high-quality total RNA extraction from adipose tissue samples. The extraction process using Trizol Reagent is based on phase separation and a series of nucleic acid precipitation methods. Nucleic acid loads of the obtained samples were fixed to a specific ng value for later steps in the study to prevent inconsistent results during qPCR.

Reverse Transcription (cDNA) Process: Oligo (dT) and Random Hexamer primers were used in the Reverse Transcription process to detect pre-mRNA and mature-mRNA structures in mRNA gene expression studies. These primers extend the mRNA chain to stabilize a structure. The Reverse Transcriptase enzyme synthesized the complementary copy of the mRNA sequence. The resulting cDNA was used as a template for standard PCR.

qPCR mRNA Gene Expression Analyses: cDNA chains created with mRNA sequences were used in relative expression analyses. Sequences of the relevant mRNA and the corresponding reference gene for each sample in the experimental and control groups were amplified using a real-time PCR machine. Data obtained from the device were evaluated using the 2-(ΔΔCt) method. The calculations used threshold values (Ct, Cp, Cq) when the fluorescence intensity crossed the threshold line during the 40 cycles.

Gene Expression Primer Design and Melting Temperatures (Tm): FAS, PPAR-γ, SREBP-1c, and ACTB gene expression levels were determined using the RT-qPCR method. RT-qPCR devices consist of a fluorometer and a thermal cycler, which detects fluorescence intensity at the end of each cycle. SYBR Green I dye, which emits fluorescence when intercalated between cDNA chains converted from RNA by reverse transcriptase, is commonly used in gene expression analyses. BrightGreen 2X qPCR MasterMix-No ROX (ABM, Vancouver, BC, Canada) and LightCycler 480 (Roche, Rotkreuz, Switzerland) were used for gene expression level determination and RT-qPCR analyses, respectively. Suitable setups were performed for each gene region, and cycle curves and Melting Curve data were examined.

Data Normalization: After device recordings, the data was normalized (Livak method). Extreme expression coefficients of genes, either too low or too high after normalization, can affect data distributions in statistical analyses. Logarithmic transformations of normalized relative gene expression levels were performed to make data distributions more symmetric, assigning equal weight to extreme and low-expression gene levels. This process largely eliminated the influence of outliers, resulting in a more straightforward dataset.

### 2.6. Statistical Analysis

The data obtained from the study was analyzed using the SPSS 24.0 software package. The normal distribution of variables was examined using analytical methods Results are presented in mean ± standard deviation (SD) values. One-way ANOVA was used for data showing normal distribution in terms of variables such as body weight, body weight gain, biochemical findings, and other quantitative data in the offspring between the control and experimental groups. Levene’s test was used to evaluate the homogeneity of variances. Tukey’s test was used as a post-hoc test for normally distributed data, and the Bonferroni test was used for those with non-normal distribution. The Kruskal–Wallis test was applied for non-parametric data. The Mann–Whitney U-test was used in pairwise comparisons, and Bonferroni correction was applied. For repeated measurements, a two-way mixed ANOVA test was used for parametric data, and the Friedman test was applied for non-parametric data. Wilcoxon test was used as a post-hoc test for the Friedman with the Bonferroni correction. The ANOVA test was applied for gene expression analysis to evaluate multiple group comparisons of groups with confirmed normal distributions. Tukey’s test was chosen as the post-hoc test. *t*-test analysis was performed for group-specific statistics for groups with confirmed normal distributions. SPSS 24.00 and GraphPad Prism programs were used to create graphs. Statistical significance was considered at a 95% confidence level with *p* < 0.05.

## 3. Results

### 3.1. Body Weights of Offspring Rats and Evaluation of Body Weight Changes

**Figure 2:**
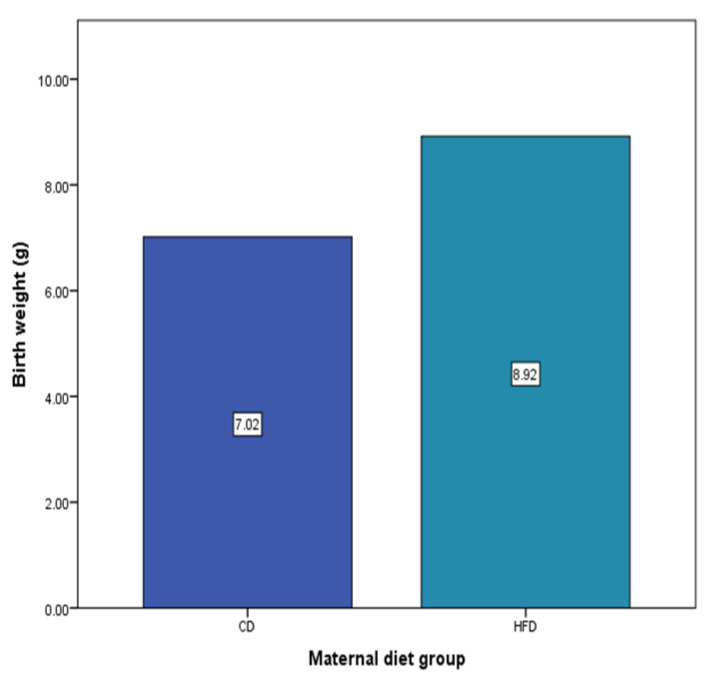
Mean Birth Weight of Offspring Rats According to Maternal Diet. CD—Control Diet, HFD—High-Fat Diet.

The birth weights of the offspring rats according to maternal nutritional status are presented in Figure 2. It was determined that the average birth weight of the offspring of the CD group mothers (7.02 ± 1.17) was lower than that of the offspring of the HFD group mothers (8.92 ± 0.55) (*p* < 0.05).

The average body weights of the offspring rats during the lactation period, based on weeks, gender, and maternal diet groups, are presented in Table 2. According to the analysis of variance in repeated measurements, both time (*p* < 0.001) and time × diet effect (*p* < 0.001) were found to be statistically significant. In measurements between weeks, the HFD-HFD group was found to be significantly different from the CD-HFD and CD-CD groups (*p* < 0.05). CD-HFD group significantly differs from HFD-CD and CD-CD groups (*p* < 0.05). When comparing body weights in the first week, the CD-CD group’s body weight was significantly lower than the HFD-HFD, HFD-CD, and CD-HFD groups (*p* < 0.05).

For the second week, the highest body weight is observed in HFD-HFD group offspring, and there is a significant difference between the CD-HFD group and HFD-HFD, HFD-CD, and CD-CD groups (*p* < 0.05). In the third week, the highest body weight is in the CD-HFD group offspring, and the lowest is in the CD-CD group offspring. According to pairwise comparisons, there are significant differences between the HFD-HFD group and CD-HFD group, HFD-CD and CD-HFD group, CD-HFD and HFD-HFD, HFD-CD, and CD-CD groups (*p* < 0.05). 

In the analysis of variance for repeated measurements, when examining body weight in male offspring rats during the lactation period, both time (*p* < 0.001) and time × diet (*p* < 0.001) effects were found to be significant. Upon examining intergroup comparisons, the CD-HFD group was found to be significantly different from the HFD-CD and CD-CD groups (*p* < 0.05). The CD-CD group of male rats had significantly lower body weight than the HFD-CD and CD-HFD groups in the first week (*p* < 0.05). In the second week, the lowest body weight in males was determined in the CD-CD group, while the highest was in the HFD-HFD group, and a significant difference was found between the CD-CD and CD-HFD groups (*p* < 0.05). Similarly, in the third week, the lowest average body weight in male offspring rats was in the CD-CD group, while the highest was in the HFD-HFD group. Intergroup comparisons found a significant difference between HFD-CD and CD-HFD groups (*p* < 0.05). 

In the analysis of variance for repeated measurements, when examining body weights in female offspring rats during the lactation period, both time (*p* < 0.001) and time × diet effects (*p* < 0.05) were found to be significant. In pairwise comparisons of body weights in the first week, the CD-CD group of female rats had significantly lower body weight than the HFD-CD and CD-HFD groups (*p* < 0.05). In the second week, the highest body weight in female rats was determined in the CD-HFD group, and pairwise group comparisons revealed significant differences between the CD-HFD group and HFD-HFD, HFD-CD, and CD-CD groups of female offspring rats (*p* < 0.05). For the third week, the highest average body weight was observed in the CD-HFD group of female offspring. Pairwise comparisons indicated significant differences between the CD-HFD group and HFD-HFD, HFD-CD, and CD-CD groups of female offspring rats (*p* < 0.05).

The average body weights of the offspring rats from the fourth week to the eighth week after weaning, based on gender and maternal diet groups, are presented in Table 3. According to the results of the two-way ANOVA test, both time (*p* < 0.001) and diet × time (*p* < 0.05) effects were found to be statistically significant. There was a statistically significant difference among weeks in all groups, with body weight gradually increasing over time (*p* < 0.001). When examining body weights in the fourth week, it was determined that the HFD-CD group had the highest body weight; however, the difference among groups was not statistically significant (*p* > 0.05). The highest average body weight from the fifth to the eighth week was observed in the HFD-HFD group, but this difference was not statistically significant (*p* > 0.05). The Friedman test results indicate that in both eight-week-old male and female offspring rats, body weights in the 4th week are significantly different from the 6th, 7th, and 8th weeks; the 5th week is significantly different from the 7th and 8th weeks, and the 6th week is significantly different from the 8th week (*p* < 0.05). When examining body weights in the fourth week, it was found that the HFD-CD group of male rats had the highest body weight, but there was no statistically significant difference among groups (*p* > 0.05). In female rats in the fourth week, the highest average body weight was observed in the CD-HFD group, but the difference among groups was not statistically significant (*p* > 0.05). In the fifth week, the highest average body weight in male offspring rats was in the CD-HFD group, but the difference was not statistically significant (*p* > 0.05). For female rats, the highest body weight was observed in the HFD-HFD group, with no significant difference among groups (*p* > 0.05). In the sixth week, the highest average body weight among male rats was in the CD-HFD group, while in female rats, the highest body weight was in the CD-CD group, with no significant difference among groups (*p* > 0.05). In the seventh week, the highest body weight in male rats was observed in the CD-HFD group, but the differences among groups were not statistically significant. The highest body weight for females was in the HFD-HFD group, with a significant difference compared to the CD-HFD and CD-CD groups (*p* < 0.05). In the eighth week, the highest average in male offspring rats was in the CD-HFD group, but there was no significant difference among groups (*p* > 0.05). The HFD-HFD group had the highest average body weight for females, and a significant difference was found when compared to the CD-HFD group (*p* < 0.05).

The evaluation of body weight changes and obesity based on maternal diet characteristics of the offspring rats is presented in Table 4. When examining weight gain, significant differences were observed among groups in three-week-old rats (*p* < 0.05). In pairwise comparisons, significant differences were found between the CD-CD group and the HFD-HFD group, as well as the HFD-CD group, and between the CD-HFD group and the HFD-HFD group, as well as the HFD-CD group (*p* < 0.05). There was a significant difference in weight gain for three-week-old male offspring rats between the HFD-CD and CD-HFD groups (*p* < 0.05). Similarly, in three-week-old female rats, a significant difference was observed between the HFD-CD and CD-HFD groups (*p* < 0.05). When examining eight-week-old offspring rats, there were no significant differences among groups regarding weight gain (*p* > 0.05). For eight-week-old male offspring rats, no significant differences were found in weight gain (*p* > 0.05). However, in eight-week-old female offspring rats, a significant difference in weight gain was observed between the CD-HFD and HFD-HFD groups (*p* < 0.05). Lee index values among three-week-old rats show significant differences (*p* < 0.05). When examining the differences between groups, a significant difference was found between the CD-CD group and the HFD-HFD and HFD-CD groups (*p* < 0.05). When the Lee index was examined based on gender in three-week-old rats, it was similar for both females and males (*p* > 0.05). There were no differences in Lee index values among eight-week-old offspring rats among groups (*p* > 0.05). The Lee index was also similar between male and female rats at eight weeks (*p* > 0.05).

### 3.2. Evaluation of Biochemical Parameters in Offspring Rats

The evaluation of biochemical parameters in the offspring rats based on weeks and maternal diet characteristics is presented in Table 5. The serum glucose level of three-week-old rat offspring was highest in the HFD-HFD group and lowest in the CD-CD group, and the difference between the HFD-HFD and CD-CD groups was statistically significant (*p* < 0.05). Among eight-week-old rats, the highest glucose level was observed in the CD-HFD group, and the lowest was in the HFD-CD group (92.1 ± 4.39 mg/dL) (*p* > 0.05). For three-week-old rat offspring, the plasma insulin level was highest in the HFD-CD group and lowest in the CD-CD group (*p* > 0.05). In eight-week-old rats, the highest insulin level was found in the HFD-CD group. When comparing groups, significant differences in insulin values were observed between the HFD-HFD and HFD-CD groups and between the HFD-CD and CD-HFD groups (*p* < 0.05). The triglyceride values for three-week-old rat offspring showed the highest level in the CD-CD group and the lowest in the HFD-HFD group (*p* < 0.05). Triglyceride levels in three-week-old rat offspring were significantly different between the HFD-HFD and CD-CD groups (*p* < 0.05). However, in eight-week-old rats, no statistically significant differences were observed between groups (*p* > 0.05). The cholesterol level of three-week-old rat offspring was highest in the CD-HFD group, but the differences in cholesterol values were not statistically significant (*p* > 0.05). In eight-week-old rats, the highest cholesterol level was observed in the CD-CD group, and the lowest was in the CD-HFD group (*p* > 0.05). The plasma leptin level in three-week-old rat offspring was highest in the CD-HFD group and lowest in the CD-CD group, with no significant difference between groups (*p* > 0.05). In eight-week-old rat offspring, leptin levels were similar among groups (*p* > 0.05). When biochemical parameters are evaluated according to gender, the differences between the groups were not found to be significant.

### 3.3. Evaluation of Adipose Tissues in Offspring Rats

**Figure 3:**
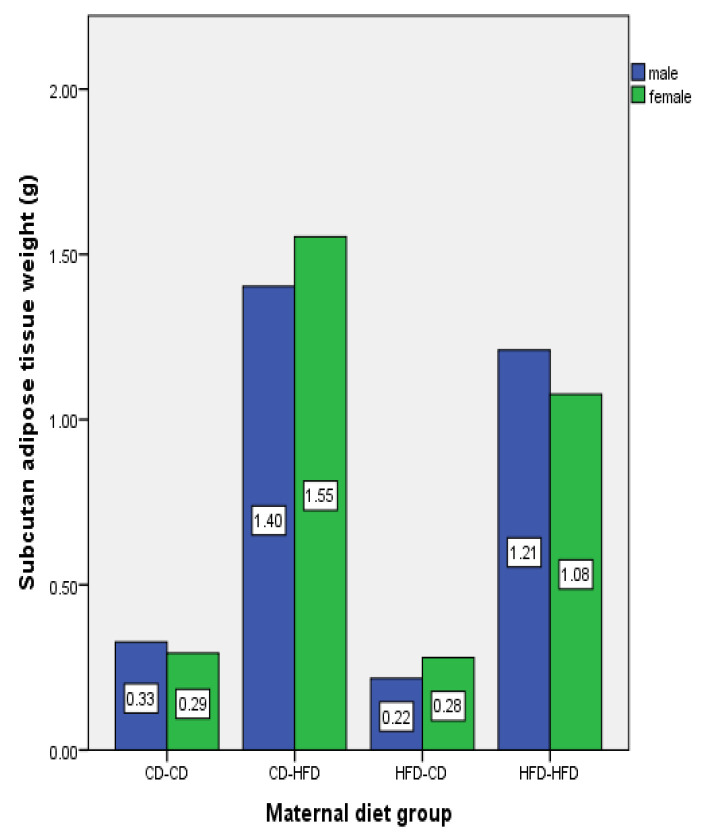
The white adipose tissue amounts of three-week-old offspring rats based on gender and maternal diet characteristics (n: 3 for female rat for each group, n: 3 for male rat for each group); CD—Control Diet, HFD—High-Fat Diet.

The amount of white adipose tissue based on maternal diet characteristics for three-week-old rat offspring is shown in Figure 3. 

Among three-week-old male rat offspring, the highest amount of adipose tissue was observed in the CD-HFD group (1.40 ± 0.06), while the lowest was in the HFD-CD group (0.22 ± 0.04). Significant differences were found between HFD-HFD (1.21 ± 0.30) and other groups, CD-HFD and HFD-CD and CD-CD group (0.33 ± 0.04) rat offspring (*p* < 0.05). When examining the adipose tissue amounts of three-week-old female rats, significant differences were observed between HFD-HFD (1.08 ± 0.12) and other groups; between CD-HFD (1.55 ± 0.11) and HFD-CD (0.28 ± 0.03) and CD-CD group (0.29 ± 0.06) offspring (*p* < 0.05).

**Figure 4:**
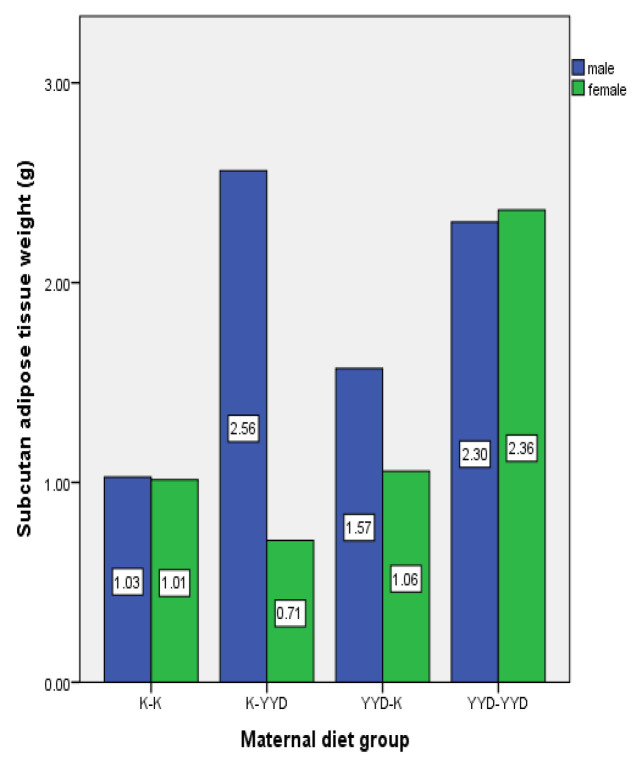
The white adipose tissue amounts of eight-week-old offspring rats based on gender and maternal diet characteristics (n: 3 for female rats for each group, n: 3 for male rats for each group). CD—Control Diet, HFD—High-Fat Diet.

The amount of white adipose tissue based on gender and maternal diet group for eight-week-old rat offspring is presented in Figure 4. When examining the adipose tissue amounts in eight-week-old male rats, it was determined that the highest amount of adipose tissue was in the CD-HFD group (2.56 ± 0.23), while the lowest was in the CD-CD group (1.03 ± 0.3). Upon comparing groups, there is a significant difference between the CD-CD group and the HFD-HFD (2.30 ± 0.82) and CD-HFD groups (*p* < 0.05). When examining the adipose tissue amounts in eight-week-old female rats, it was found that the highest amount was in the HFD-HFD group (2.36 ± 0.62), and the lowest was in the CD-HFD group (0.71 ± 1.82). In pairwise group comparisons, a significant difference was found between the HFD-HFD and CD-HFD groups (*p* < 0.05).

### 3.4. Effects of Maternal Diet on Gene Expression in Offspring Rats

The fold change in gene expression levels of FAS, SREBP-1c, and PPAR-γ genes according to week, maternal diet and gender is presented in Table 6. Three-week-old rats exhibited the highest relative gene expression level of the FAS and SREBP-1c gene in the CD-CD group and the lowest in the HFD-CD group (*p* > 0.05). The relative gene expression levels of PPAR-γ were highest in CD-CD and lowest in CD-HFD group (*p* > 0.05). At eight weeks, FAS expressions were highest in CD-HFD and lowest in CD-CD (*p* > 0.05). SREBP-1c expression was highest in HFD-CD and lowest in CD-HFD (*p* > 0.05). PPAR-γ expression was highest in HFD-CD and lowest in CD-HFD (*p* > 0.05). In both 8-week-old and 3-week-old rats, when examining the differences between groups based on gender, statistically significant distinctions were not found (*p* > 0.05).

### 3.5. Maternal-Diet-Related Histological Analyses in Adipose Tissues of Offspring Rats

**Figure 5:**
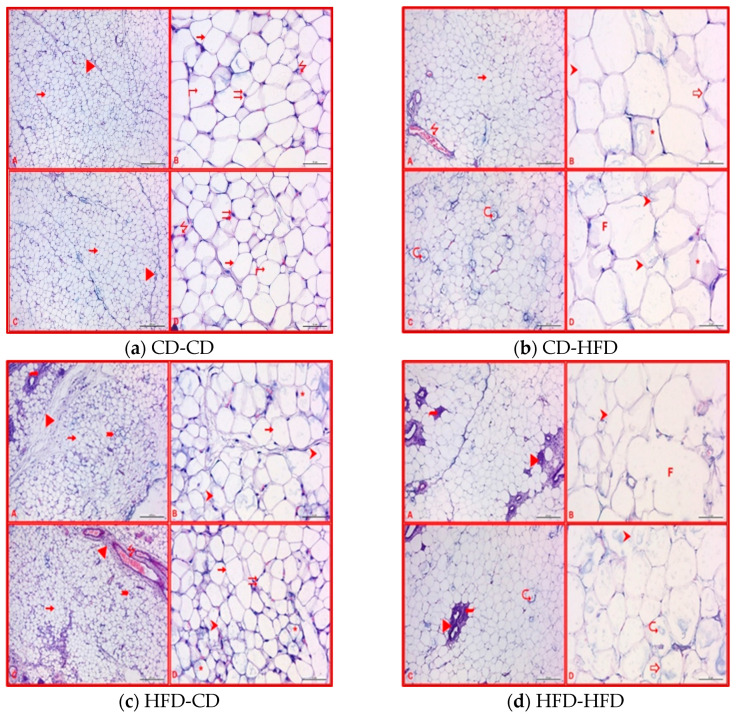
Adipose tissue examinations of 3-week-old rats for each group. (**a**) CD-CD: ⭢—white adipose tissue cells, ▶—connective tissue, ⇉—nucleus, ⭍ —blood vessels and ⮣—adipocytes with a homogeneous appearance (**b**) CD-HFD: 3-week-old rats ⭢—white adipose tissue cells, ⭍ —blood vessels, *—indicates adipocytes with a heterogeneous appearance, ⮞—deformed adipocytes, ➩—undulation, ⮎—myeline-like figure ve F—fusion (**c**) HFD-CD: ⭢—white adipose tissue cells, ▶—increased density of connective tissue, ⇉—nucleus, ⭍ —dilated blood vessel, ⮫—infiltration, 🢚—brown adipose tissue cells, *—heterogeneous appearance in adipocytes and ⮞—deformed adipocytes (**d**) HFD-HFD: ▶—increased density of connective tissue, ⮫—infiltration, ⮞—deformed adipocytes, ➩—undulation, ⮎—myelin-like figure ve F—fusion (A, B—Female, C, D—Male) (Hematoxylin and Eosin ×100 [A, C], ×400 [B, D]). CD—Control Diet, HFD—High-Fat Diet.

Histological examinations of the adipose tissues in three-week-old offspring using Hematoxylin and Eosin staining are illustrated in Figure 5. Histological examinations of the adipose tissues in CD-CD three-week-old offspring using Hematoxylin and Eosin staining are illustrated in Figure 5a. The microscopic analyses were conducted at both low and high magnifications. The adipose tissue’s white adipocyte cells and the occasional fine connective tissue elements in between were observed to maintain a normal appearance. The adipose tissue cells were characterized by uniform sizes, smooth contours, and univacuolar flat cell nuclei located at the cell periphery. Additionally, normal-sized blood vessels were intermittently observed in the connective tissue. The homogeneous appearance of adipocytes suggested an absence of lipid content alterations. Microscopic examinations in CD-HFD three-week-old offspring are presented in Figure 5b. Both low- and high-magnification images are included in the illustration. Compared to the other groups, this group had a generally more deformed appearance. While dilated vessels were occasionally prominent, the severe increase in connective tissue observed in the HFD-CD group was not identified in this group. Some adipocytes were noted to contain myelin-like figures, indicative of lipid content alterations, displaying an overall heterogeneous appearance. Significant deformation in cells and a resultant undulating pattern were observed. Adipocytes undergoing fusion were discerned in some areas, and an increase in volume was noted in adipocytes compared to other groups. Microscopic examinations of the adipose tissues in HFD-CD three weeks offspring are presented in Figure 5c. The most notable findings in this group were characterized by an increase in the amount of connective tissue, infiltration, dilation of blood vessels, and the presence of cells belonging to brown adipose tissue in some areas. The brown adipose tissue observed in this group was considered to regulate inflammation by reducing pro-inflammatory cells in infiltration areas. Deformation and a heterogeneous appearance were observed in some adipocytes in this group. Deformed adipocytes showed indistinct cell nuclei. Microscopic examinations of adipose tissues in HFD-HFD three-week-old offspring are presented in Figure 5d. This group exhibited the most degenerative findings among the three-week-old groups. An increase in connective tissue, believed to be associated with HFD during pregnancy, was observed in this group. Infiltration was identified in these areas. While myelin-like figures were occasionally observed in adipocytes, it was noted that adipocytes underwent fusion, losing cell integrity and displaying a degenerative appearance. Distorted shapes and undulation were also observed in adipocytes in this group. When adipocytes were evaluated volumetrically, it was discerned that there was no pronounced increase in volume compared to the previous group, excluding fused adipocytes. Despite applying a HFD during pregnancy and lactation, a consistent feeding profile in this group, as opposed to transitioning from a standard diet to a HFD as in the previous group, was considered a possible explanation for this observation.

**Figure 6:**
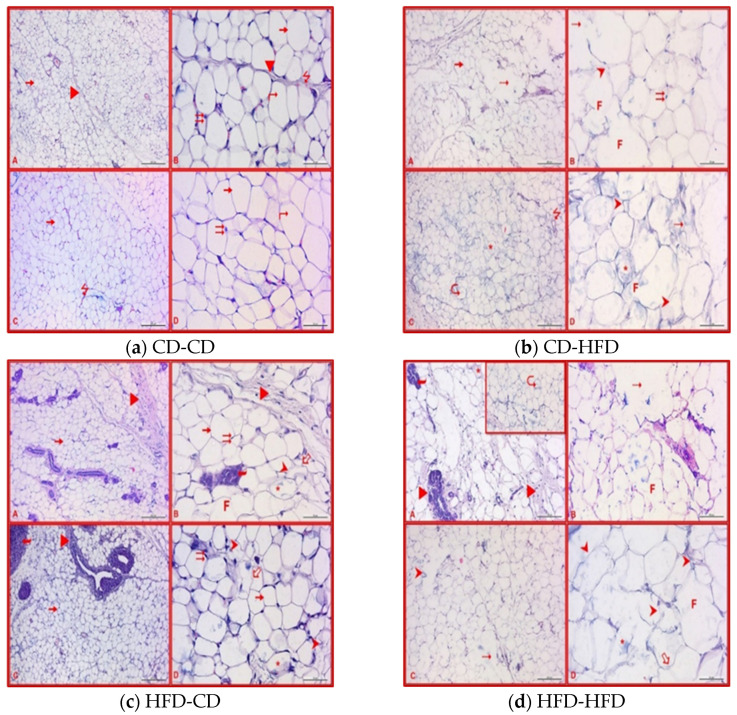
Adipose tissue examinations of 8-week-old rats. (**a**) CD-CD: ⭢—white adipose tissue cells, ▶—connective tissue, ⇉—nucleus, ⭍ —blood vessels and ⮣—indicates adipocytes with a homogeneous appearance (**b**) CD-HFD: ⭢—white adipose tissue cells, ⇉—nucleus, ⭍ —blood vessel, *—adipocytes with a heterogeneous appearance, ⮞—deformed adipocytes, ⮎—myeline-like figure, ⇢ —areas with lost tissue integrity and F: fusion (**c**) HFD-CD: ⭢—white adipose tissue cells, ▶—increased density of connective tissue, ⇉—nucleus, ⮫—infiltration, *—heterogeneous appearance in adipocytes, F—fusion, ➩—undulation and ⮞—deformed adipocytes (A, B—Female, C, D—Male) (Hematoxylin and Eosin ×100 [A, C], ×400 [B, D]) (**d**) HFD-HFD: ▶—increased density of connective tissue, ⮫—infiltration, ⮞—deformed adipocytes, ➩—undulation, ⮎—myelin-like figure, *—heterogeneous appearance in adipocytes and F—fusion (A ve A-Inset, B—Female, C, D—Male) (Hematoksilen Eozin ×100 [A ve A-Inset, C], ×400 [B, D]). CD—Control Diet, HFD—High-Fat Diet.

In the microscopic examinations conducted on adipose tissue of eight-week-old using Hematoxylin–Eosin staining (Figure 6). Adipose tissue of CD-CD eight-week-old rats, both at low and high magnifications, are shown in Figure 6a. The adipose tissue exhibited a normal histological appearance, similar to the three-week control group. In the microscopic examinations conducted on adipose tissues of CD-HFD eight-week-old using Hematoxylin–Eosin staining, both at low and high magnifications, (Figure 6b), revealed the loss of tissue integrity in certain areas. Fusion, myelin-like figures, and volumetric increase were observed in adipocytes. Cells that were completely deformed and had indistinct boundaries were also noteworthy, excluding fused adipocytes. HFD-CD 8-week-old offspring underwent microscopic examinations of their adipose tissues. As shown in Figure 6c, the most prominent finding, similar to the three-week group, was an increase in the density of connective tissue and infiltration. Brown adipose tissue presence was observed in some subjects again. Heterogeneous appearance, deformation, undulation, and occasional fusion were identified in adipocytes. Microscopic examinations of HFD-HFD 8-week-old offspring adipose tissues, as depicted in Figure 6d, showed an increase in connective tissue, presumed to be associated with a high-fat diet during pregnancy. Deformed and heterogeneous adipocytes were identified. Myelin-like structures were also present in this group. Areas with compromised tissue integrity were noted, and fusion of adipocytes was observed in this group as well.

When considering the differences between female and male subjects in all histological findings, it was observed that some female subjects exhibited a more degenerative appearance compared to male subjects. However, considering all subjects, it was determined that the applied HFD did not create a significant difference in the reflection of adipose tissues in male and female subjects. 

According to histological findings, the most degenerative changes in adipose tissue among all groups were observed in eight-week-old subjects. When groups with transitions between HFD and CD were evaluated, although significant degeneration was observed in groups where an HFD was applied during pregnancy and a CD during lactation, it was distinguished that groups where a CD was applied during pregnancy and an HFD during lactation exhibited more degenerative features in adipose tissue. However, it was concluded that the most degenerative findings occurred as a result of an HFD during both pregnancy and lactation periods (Table 7).

## 4. Discussion

The prenatal and postnatal maternal environment significantly influences obesity and contributes to the transfer of metabolic dysfunctions between generations [23,24,25,26]. Although the effects of maternal HFD in the prenatal and postnatal periods are more clear, their relative effects in separate developmental periods still need to be clarified. However, the concept of lactational programming, which has been put forward in recent years, and therefore the lactation period, is a crucial window of opportunity for the health outcomes of the offspring [23]. This study aimed to assess the effects of HFD during only pregnancy and/or lactation, without intervening in maternal dietary conditions before pregnancy and after the offspring’s weaning. Nonetheless, most studies have utilized animal models examining long-term paradigms. Researchers predominantly applied HFD during pregnancy and lactation, along with varying periods before maternal pregnancy, and fed the offspring with HFD for different durations (8–36 weeks) after the lactation period [27]. Therefore, most studies in the literature cover the long-term changes of maternal HFD applied for a long time to the offspring. In order to develop targeted interventions, it is critical to ascertain the specific impact of maternal physiological status throughout periconception, pregnancy, and lactation on metabolic outcomes in progeny.

The effects of maternal HFD on fetal growth are contradictory [5,27,28,29]. In a rat model administered HFD before and during pregnancy and lactation, newborn offspring of obese mothers were found to be normotrophic [27]. A meta-analysis involving birth weight data from 57 studies in males and 14 studies in females following exposure to maternal diet showed no overall effect of maternal diet on birth weight [28]. However, considering species, maternal HFD exposure increased birth weight in male mice, while an opposite trend was found in male rats. Although not reaching statistical significance, a similar trend was observed in female mice and rats. No significant effect was found in 14 studies that reported male and female offspring data together [29]. No effect was found on the birth weight of male or female offspring of mothers fed a Western diet throughout pregnancy and lactation [5]. In a study with a cross-fostering design, the weights of newborn offspring of pregnant mice fed HFD at 0.5 days after birth were significantly higher than those of newborns of pregnant mice fed a diet [30]. Similarly, in this study, the birth weights of the offspring born to HFD group mothers were found to be higher (*p* < 0.05).

In one study, rats were given either HFD or CD three weeks before mating. Compared with the offspring of CD group mothers, the offspring of HFD group mothers showed a significant increase in body weight on postnatal days one, five, and ten. Gender did not have an impact on body weight. Increased body length, weight, and adiposity were observed in the offspring of maternal HFD groups on postnatal day 10 [31]. In another study, the offspring of mothers fed an HFD gained more body weight than the offspring of CD mothers on postnatal day 7, and this difference persisted throughout lactation [32]. In another study, total body weight gain during lactation was 30% higher in male offspring and 35% higher in female offspring born and fed to mothers fed a Western diet than in female offspring born to mothers fed a CD diet [33]. In one study, newborns born to mothers exposed to HFD exhibited rapid body weight gain during lactation, an essential period of adipose tissue development in rodents. The increased body weight in male offspring continued until three months of age [27]. A meta-regression analysis revealed maternal HFD exposure was associated with increased body weight at weaning in both male and female offspring [29]. Similarly, in this study, the HFD-HFD group at weaning had significantly higher body weight than other groups (*p* < 0.05). The weight differences between groups at weaning could be the HFD applied during lactation, potentially leading to hyperphagia and increased milk consumption. Purcell and colleagues [34] showed in their study that the offspring of mothers fed an HFD consumed more breast milk. Another study discovered that offspring who were exposed to a maternal HFD exhibited larger size, suggesting that they may have more energy needs compared to control offspring. As a result, these offspring may require a greater amount of milk to fulfill these energy requirements [31]. Another study also showed that mothers exposed to HFD spent more time breastfeeding and caring for their babies, and their offspring’s milk consumption increased [34]. However, hyperphagia and physical inactivity were also documented in three-week-old progeny of obese mothers, as reported by Samuelsson et al. [35]. Therefore, increased milk intake also contributed to the results. In a study in which mothers were given HFD during the lactation period, it was reported that the breast milk composition of mother rats in the HFD group contained higher fat and energy than the milk of mothers in the control group [36]. Purcell et al. [34] determined that the energy and fat content of the milk of rats fed HFD on the tenth postnatal day was higher. 

Perinatal nutrition supply disruptions can cause persistent alterations in adipose tissue growth’s quantity and functional attributes [37,38]. Limited knowledge exists regarding programming mechanisms that could elucidate prolonged disturbances in adipogenesis and white adipose tissue (WAT) metabolism in offspring born to mothers with obesity. Specifically, the occurrence of obesity in mothers during conception has led to an increase in the formation of fat cells and the production of fat molecules from the fetal stage to adulthood. This has resulted in a greater amount of white adipose tissue and larger fat cells [39]. Overnutrition/HFD during lactation and/or post-weaning periods accelerates growth, deteriorating adipogenesis, and lipogenesis programming [21,38]. Maternal diet can significantly affect energy expenditure or metabolic outcomes in offspring, while their intake of maternal milk and post-weaning diet also notably influences body weight [40]. The study found that while maternal HFD didn’t affect the birth weight of offspring, HFD group offspring showed increased body weight and adiposity compared to the control group during weaning [17]. Offspring from mothers fed HFD during weaning showed significantly higher body weight than mothers fed CD [41]. It was observed that the early increase in body weight of HFD offspring during weaning rapidly normalized within two weeks after transitioning to a CD following the cessation of high-fat intake through lactation. Additionally, it was found that the increase in fat mass of offspring nursed by mothers from the CD group and those from mothers from the HFD group was significantly higher than that of 14-week-old mice born to and nursed by CD mothers. Similarly, the fat mass increase of offspring from HFD mothers and nursed by HFD mothers was higher than the 8-week-old mice born to CD mothers but nursed by HFD mothers. Moreover, the fat mass increase of offspring from CD mothers and nursed by HFD mothers was higher than the 16-week-old offspring born to and nursed by CD mothers. In female offspring, mild effects of maternal obesity induced by HFD were observed on body weight, energy intake, and the ratio of fat mass to body weight compared to male offspring [30]. HFD offspring showed a significant increase in fat mass percentage relative to lean mass [31]. Consistently, the ratios of fat pad weight and individual fat weight to body weight were higher in HFD offspring, indicating higher adiposity in adulthood [32]. Inguinal and epididymal adipose tissue adipocyte sizes were larger in HFD offspring than in the control group offspring [24]. In our study, mothers maintaining a normal diet before pregnancy and not being obese limited the obesity status of the offspring in our study. Although the Lee index values of the rats were different at 3 weeks, this difference was not observed when they were eight weeks old. These results in offspring fed CD after the weaning period suggest that the effects of maternal HFD can be compensated by switching to a normal-fat diet after weaning. Still, making a clear interpretation is impossible because the follow-up period is limited to eight weeks.

While white adipose tissue has a lesser function in glucose uptake than skeletal muscle, visceral adipose tissue is especially significant in developing insulin resistance. Many studies investigating the effects of maternal HFD have observed that diets given starting from the pre-pregnancy period, especially in female offspring, lead to increased adipose tissue formation [42,43]. The most significant difference between these studies and ours is the absence of maternal obesity in our study. In our study, we investigated the effects of maternal diet composition, specifically during pregnancy and/or lactation, on offspring without the presence of maternal obesity. Despite mothers not being obese in our study, both the amount and histology of adipose tissue in the offspring were influenced by their mother’s diet. During weaning, HFD offspring showed larger deposits were accompanied by hypertrophy in the white adipocytes in HFD offspring [32]. In our study, the most degenerative changes were observed in the eight-week-old HFD-HFD group when a histological examination of adipose tissues was performed. Groups transitioning from CD during pregnancy to HFD during lactation also showed degenerative signs in adipose tissues. This outcome suggests that the most significant impact is attributed to HFD exposure during pregnancy and lactation, but exposure only during lactation could still contribute to more adiposity. In addition, this might stem from inconsistencies in maternal nutrient provisions pre- and post-birth. Earlier research also implies that female offspring might be more susceptible to diet-induced obesity [44,45]. Male offspring at four and twelve weeks of age that were exposed to maternal obesity exhibited greater perirenal fat mass than their female counterparts in a study that utilized cafeteria or CD. Female offspring, in contrast, had greater gonadal fat. Gonadal adiposity was 26% greater in female offspring at 12 weeks of age that were subjected to maternal obesity during lactation, as compared to those that were exposed to a control diet [33]. These sex differences may provide important information about which tissues the metabolic effects of obesity are concentrated in and how they differ between different sexes [33]. Although some female subjects showed a more degenerative appearance than male subjects in the histological findings, the applied maternal HFD did not significantly differentiate the reflections in the adipose tissues of male and female offspring. 

High-fat diets change DNA methylation, and DNA methylation influences the mRNA level of genes involved in lipid metabolism [46]. Activation of PPAR-γ increases lipoprotein lipase activity in adipose tissue, leading to higher fatty acid distribution and storage of triglycerides in adipose tissue, thereby resulting in lower levels of free fatty acids in the plasma and increased insulin sensitivity [47]. Increased mRNA expression of PPAR-γ in fetal/neonatal fat depots has been reported in offspring of mother rats fed a junk food diet during both pregnancy and lactation, particularly in late pregnancy [48]. In fetal sheep, an increase in PPAR-γ mRNA expression in response to maternal overnutrition has also been associated with the upregulation of key lipogenic genes, including lipoprotein lipase, in the same fat depots [49]. SREBP-1a and SREBP-1c are responsible for controlling the activity of genes that play a role in the production of fatty acids, including acetyl-CoA carboxylase, fatty acid synthase, and sterol-CoA desaturase [50]. Obesity-related problems have been linked to the persistent activation of SREBP-1c caused by excessive eating. Moreover, persistent activation of SREBP-1c has been found to cause elevated triglyceride synthesis and lipid deposits in multiple organs, including the liver and pancreas. In a study, the mRNA level of FAS, which supports fatty acid biosynthesis, increased significantly with post-weaning HFD intake in offspring. At the same time, the mRNA levels of SREBP-1c, which is a transcription factor for FAS, in the groups fed HFD after weaning following maternal HFD and maternal CD application were found to be significantly lower than the group that received maternal CD and continued to be fed CD after weaning [36]. In this study, FAS-relative gene expression levels of both three-week-old and eight-week-old rats were found to be similar between the groups (*p* > 0.05). Similarly, the FAS relative gene expression level of both three-week-old male and female rats does not significantly differ between the groups (*p* > 0.05). A study revealed that the expression of SREBP-1c mRNA was elevated in offspring who were exposed to maternal obesity prior to birth, in contrast to offspring who were subjected to a standard diet at the same period [33]. This suggests that increased PPAR-γ expression may be associated with adipocyte hypertrophy in offspring. These results indicate that maternal HFD during lactation promotes lipid uptake into adipocytes, lipid and carbohydrate metabolism in adipocytes, and inhibits lipid secretion into the blood. In addition, the increase in PPAR-γ may be due to the fact that the offspring are fed with high-fatty acid and high-energy milk during lactation, leading to high non-esterified fatty acids and insulin levels in these offspring [51]. In a study, maternal HFD exposure exhibited increased SREBP1-c and FAS and decreased PPAR-γ mRNA levels [27]. Conversely, the female offspring showed few metabolic differences when exposed to a high-fat diet during pregnancy. Therefore, maternal obesity and increased growth during lactation cause offspring to have a greater amount of body fat through changes in gene expression in the visceral fat tissue, which varies depending on the sex of the offspring [52]. In our study, SREBP-1c and PPAR-γ gene expression levels in male and female rats were found to be similar (*p* > 0.05). It was determined that the relative gene expression levels of SREBP-1c and PPAR-γ in both three-week-old and eight-week-old rats were similar between the groups (*p* > 0.05). It is possible that the most important reason for the difference in this study is that CD was fed instead of HFD after weaning. In addition, the fact that the mothers were not administered HFD during the pre-pregnancy period may have affected the results. These results suggest that feeding with HFD after the weaning period may affect gene expression in lipid metabolism more than feeding with HFD during the maternal period. 

Insulin plays a role in controlling eating behavior by regulating satiety signals. Decreased insulin sensitivity contributes to increased food consumption, impulsive eating and obesity [53]. In addition, insulin is also required for adipocyte differentiation and development and is an activator of PPAR-γ [53]. In a study conducted to observe the effects of maternal HFD, hyperglycemia was not observed. No changes in serum insulin, adiponectin or free fatty acids were detected, despite higher adiposity in the HFD group offspring, suggesting insulin resistance [17]. In a study, the serum glucose level in the HFD-HFD group was significantly higher than the CD-CD group, indicating that HFD consumption in offspring and mothers affects the serum glucose level in the offspring [36]. Similarly, in our study, the glucose levels of three-week-old rat offspring were found to be the highest in the HFD-HFD group and the lowest in the CD-CD group (*p* < 0.05). But among eight-week-old rats, this difference was not significant (*p* > 0.05). Krause et al. [52] showed that although a difference was detected in body weight at six weeks of age, no significant difference was observed in adiposity, fasting blood glucose and insulin levels when the offspring were 26 weeks old. Male mice born to HFD mothers and nursed by HFD mothers exhibited significantly increased HOMA-IR values compared to mice born to CD mothers and nursed by CD mothers at both 12 and 24 weeks of age, with no significant changes in HOMA-IR detected at 12 weeks of age among the other three groups, but significant changes at 24 weeks of age, differences have been identified [30]. In another study, no significant difference was shown in fasting insulin levels and glucose tolerance in the 12-week-old male offspring of mothers exposed to HFD only during pregnancy [54]. In our study, plasma insulin levels of three-week-old rat offspring were found to be the highest in the HFD-CD group and lowest in the CD-CD group (*p* > 0.05). In eight-week-old rats, the highest insulin levels were identified in the HFD-CD group (*p* < 0.05). In this context, it can be considered that transitioning to a CD during lactation and continuing this diet post-lactation may enhance insulin sensitivity. However, the reasons for the observed differences in metabolic outcomes among studies may be attributed to the duration of the applied maternal HFD (especially whether it was applied before pregnancy) and the period during which it was implemented (during pregnancy and/or lactation), as generally seen in conducted studies.

In individuals with increased adiposity, the level of leptin released from adipose tissues into the circulation increases [55]. Additionally, adipocyte hypertrophy has been reported to cause an increase in serum leptin levels [56]. In a study, triglyceride and leptin levels of both 12- and 24-week-old male and female rat offspring of mothers exposed to HFD during the lactation period were found to be significantly higher than controls. Moreover, notable disparities between sexes were observed in triglyceride levels at 12 and 24 weeks of age, as well as in leptin levels at 24 weeks of age. The offspring of mothers who consumed HFD during nursing experienced elevated and protracted fluctuations in leptin levels, regardless of the mother’s nutritional condition during pregnancy [57]. In this study, plasma leptin levels of three- and eight-week-old female and male offspring were found to be similar between the groups (*p* > 0.05). In a study in which a maternal HFD or CD diet was applied during the lactation period and HFD or CD was applied to the offspring after weaning, serum triglyceride and total cholesterol levels increased with post-weaning HFD intake in the offspring. However, these levels did not differ significantly depending on the mother’s diet during lactation. Therefore, administration of HFD during lactation was found to have little effect on serum lipid parameters in offspring [36]. In a study in which CD or HFD was applied during pregnancy and lactation, maternal HFD increased visceral adiposity and serum cholesterol in newborn rats [58]. In our study, when the plasma triglyceride values of three-week-old baby rats were examined, it was determined that the highest value was in the CD-CD group, and the lowest value was in the HFD-HFD group (*p* < 0.05). When eight-week-old rats were examined, it was determined that the difference between the groups was not statistically significant (*p* > 0.05). Cholesterol values of three- and eight-week-old female and male offspring were found to be similar between the groups (*p* > 0.05). However, the fact that HFD application in the post-weaning period rather than maternal HFD application is more important in terms of blood lipids may have ensured that the blood lipids of the eight-week-old offspring were similar in this study. When the incompatible results are compared with our study, the shorter HFD exposure period in this study and the feeding of the offspring with CD after weaning may have caused the difference in the results. Although consumption of a post-weaning CD may show ameliorative effects, it may not be sufficient to ameliorate many of the changes in hypothalamic programming caused by maternal nutrition. 

Some limitations in our study can be mentioned. In interpreting the data obtained, examining breast milk along with the findings may contribute to explaining possible mechanisms. Among the difficulties encountered in the study was a delay in the start of the supply of feed imported from abroad due to the COVID-19 pandemic. Another issue that needs to be considered in interpreting the data is gender-specific effects. Increasing the number of male and female rats in this study can strengthen the study. The use of a standard feed with different micronutrients and vitamin concentrations as a control diet instead of a specially produced control diet is among the limitations of the study. One of the important points about animal studies is their applicability to humans. The impact of maternal diet on offspring metabolism has been shown, although it remains uncertain how applicable these findings are to humans.

## 5. Conclusions

Adipose tissue programming and nutritional status may encounter significant alterations during pregnancy and the early postpartum period. As a result of this study, it was determined that the blood glucose, triglyceride, adipose tissue quantity and histology of the offspring were affected by the maternal diet in the early period, especially during the weaning period. Although the effects of HFD are stronger, especially during both pregnancy and lactation periods, it has been determined that HFD exposure only during the lactation period also increases adiposity and increases the number of adipocytes. Based on the findings obtained, it is thought that the effect of the diet during the lactation period and the maternal diet during pregnancy is quite high in terms of the effects on the offspring rats. In addition, in our study, continuing the normal-fat diet of the offspring after lactation may have prevented the emergence of negative effects due to maternal diet during pregnancy and/or lactation to some extent. However, it is quite remarkable that the changes in the histological dimension continued in eight-week-old rats. It also suggests that the effects of maternal diet may be reflected in the long term. In addition, conducting studies involving more male and female offspring will enable a clearer evaluation of the differences in the effect of maternal diet on gender problems. In future studies on this subject, studies such as evaluating the effects of specific macro and micronutrients, examining the function of the placenta, and evaluating the microbiota will be guiding in elucidating the mechanisms.

## Figures and Tables

**Table 1:** Diet characteristics of mother rats during pregnancy and lactation.

Group	Pregnancy Period	Lactation Period
Group 1: CD *-CD	Control Diet	Control Diet
Group 2: CD-HFD **	Control Diet	High-Fat Diet
Group 3: HFD-CD	High-Fat Diet	Control Diet
Group 4: HFD-HFD	High-Fat Diet	High-Fat Diet

* CD—Control Diet, ** HFD—High-Fat Diet.

**Table 2:** Weekly body weights of offspring during the lactation period according to maternal diet.

Maternal Diet Group	Body Weights of Offspring			
	End of 1st Week	End of 2nd Week	End of 3rd Week	
	$\bar{x}$ ± SD	$\bar{x}$ ± SD	$\bar{x}$ ± SD	*p* *
CD-CD (n: 12)	11.7 ± 1.74 ^ac^	23.0 ± 3.20	45.9 ± 2.34	<0.001 ***
CD-HFD (n: 12)	16.6 ± 0.88 ^ab^	29.5 ± 2.14 ^abcd^	55.0 ± 1.92	<0.001 ***
HFD-CD (n: 12)	16.4 ± 1.45 ^a^	25.5 ± 2.43 ^c^	50.7 ± 4.28	<0.001 ***
HFD-HFD (n: 12)	15.8 ± 1.14 ^a^	30.2 ± 4.11 ^a^	54.4 ± 2.27	<0.001 ***
*p* **	<0.001 ***	<0.001 ***	<0.001 ***	
Male				
CD-CD (n: 6)	12.4 ± 1.71 ^a^	25.3 ± 2.60 ^a^	46.4 ± 1.28	<0.001 ***
CD-HFD (n: 6)	16.0 ± 0.45 ^b^	28.4 ± 2.69 ^b^	54.5 ± 1.34 ^a^	<0.001 ***
HFD-CD (n: 6)	17.0 ± 1.89 ^b^	27.2 ± 2.33	52.7 ± 3.38 ^b^	<0.001 ***
HFD-HFD (n: 6)	16.7 ± 0.65 ^b^	32.2 ± 4.90	55.0 ± 3.00	<0.001 ***
*p* **	0.022 ***	0.022 ***	0.04 ***	
Female				
CD-CD (n: 6)	10.9 ± 1.72 ^a^	20.6 ± 1.60 ^b^	45.3 ± 3.35 ^b^	<0.001 ***
CD-HFD (n: 6)	17.3 ± 0.58 ^b^	30.6 ± 0.86 ^a^	55.6 ± 2.55 ^a^	<0.001 ***
HFD-CD (n: 6)	15.8 ± 0.69 ^b^	23.8 ± 1.01 ^b^	48.7 ± 4.75 ^b^	<0.001 ***
HFD-HFD (n: 6)	15.0 ± 0.87	28.2 ± 2.61 ^b^	53.9 ± 1.73 ^b^	<0.001 ***
*p* **	<0.001 ***	<0.001 ***	<0.001 ***	

* In repeated measurements, the ANOVA test, ** One-way ANOVA test, *** *p* < 0.05, ^a^, ^b^, ^c^, ^d^ Different letters indicate significant differences between groups for each column (*p* < 0.05), while the same letters indicate non-significant differences (*p* > 0.05), CD—Control Diet, HFD—High-Fat Diet.

**Table 3:** Weekly post-weaning body weights of eight-week-old offspring rats according to gender and maternal diet.

Maternal Diet Group	Post-Weaning Body Weights of Eight-Week-Old Rats					
	End of 4th Week	End of 5th Week	End of 6th Week	End of 7th Week	End of 8th Week	
	$\bar{x}$ ± SD	$\bar{x}$ ± SD	$\bar{x}$ ± SD	$\bar{x}$ ± SD	$\bar{x}$ ± SD	*p* *
CD-CD (n: 6)	69.5 ± 6.76	92.0 ± 5.64	120.3 ± 8.18	133.4 ± 6.66	159.0 ± 11.64	<0.001 ^†^
CD-HFD (n: 6)	77.5 ± 3.23	106.8 ± 18.11	141.3 ± 35.10	169.4 ± 48.36	200.0 ± 61.37	<0.001 ^†^
HFD-CD (n: 6)	78.2 ± 6.03	105.0 ± 14.90	129.5 ± 20.53	163.0 ± 22.60	201.8 ± 32.71	<0.001 ^†^
HFD-HFD (n: 6)	76.7 ± 7.07	107.7 ± 13.34	144.1 ± 9.98	181.5 ± 24.28	226.5 ± 39.11	<0.001 ^†^
*p* **	0.069	0.199	0.221	0.061	0.061	
Male ***						
CD-CD (n: 3)	73.7 ± 6.29	95.6 ± 2.70	123.2 ± 10.89	133.9 ± 10.01	165.3 ± 13.57	<0.001 ^†^
CD-HFD (n: 3)	79.4 ± 2.65	118.6 ± 19.85	168.3 ± 29.83	209.6 ± 30.98	255.6 ± 10.40	<0.001 ^†^
HFD-CD (n: 3)	81.7 ± 7.01	111.5 ± 19.48	138.6 ± 26.50	179.3 ± 16.01	219.0 ± 33.15	<0.001 ^†^
HFD-HFD (n: 3)	79.1 ± 8.34	100.1 ± 13.07	143.3 ± 15.30	188.3 ± 30.66	232.0 ± 47.75	<0.001 ^†^
*p* ****	0.459	0.361	0.282	0.066	0.066	
Female ***						
CD-CD (n: 3)	65.3 ± 4.72	88.4 ± 5.81	145.0 ± 3.60	132.9 ± 3.16 ^b^	152.6 ± 5.85 ^a^	<0.001 ^†^
CD-HFD (n: 3)	75.6 ± 2.85	94.9 ± 1.90	120.5 ± 10.21	129.1 ± 5.34 ^c^	144.3 ± 3.51 ^a^	<0.001 ^†^
HFD-CD (n: 3)	74.8 ± 2.43	98.6 ± 7.24	114.4 ± 3.50	146.6 ± 14.84 ^a^	184.6 ± 26.31 ^a^	<0.001 ^†^
HFD-HFD (n: 3)	74.4 ± 6.23	115.2 ± 10.12	117.4 ± 4.84	174.8 ± 19.93 ^a^	221.0 ± 38.11 ^b^	<0.001 ^†^
*p* ****	0.147	0.055	0.077	0.039 ^†^	0.026 ^†^	

* In repeated measurements ANOVA test ** One-way ANOVA test, *** Friedman test, **** Kruskal Wallis test, ^†^ *p* < 0.05, ^a^, ^b^, ^c^ Different letters indicate significant differences between groups for each column (*p* < 0.05), while the same letters indicate non-significant differences *p* > 0.05, CD—Control Diet, HFD—High-Fat Diet.

**Table 4:** Evaluation of body weight change and obesity of offspring according to their maternal diet characteristics, gender, and weeks of age.

	CD-CD	CD-HFD	HFD-CD	HFD-HFD	
	$\bar{x}$ ± SD	$\bar{x}$ ± SD	$\bar{x}$ ± SD	$\bar{x}$ ± SD	*p*
3-week-old rats (n: 12)					
BWG **	51.2 ± 4.97 ^ac^	52.2 ± 4.50 ^ac^	37.6 ± 2.10 ^b,c^	38.8 ± 2.36 ^b,c^	<0.001 *
Lee index **	0.26 ± 0.004 ^a^	0.28 ± 0.225 ^a^	0.30 ± 0.008 ^a,b^	0.29 ± 0.008 ^a,b^	0.001 *
Male (n: 6) **					
BWG-3w	51.7 ± 11.74 ^a^	57.0 ± 14.15 ^b^	40.1 ± 4.84 ^bc^	42.1 ± 3.97 ^a^	0.021 *
Lee index-3w	0.27 ± 0.005	0.27 ± 0.02	0.30 ± 0.01	0.29 ± 0.01	0.190
Female (n: 6) **					
BWG-3w	42.3 ± 7.71 ^a^	49.9 ± 6.68 ^b^	37.5 ± 4.61 ^bc^	42.5 ± 7.25 ^d^	0.033 *
Lee index-3w	0.27 ± 0.000	0.29 ± 0.011	0.30 ± 0.005	0.29 ± 0.005	0.056
8-week-old-rats (n: 6)					
BWG **	152.9 ± 11.72	189.9 ± 58.9	191.9 ± 31.30	214.0 ± 36.89	0.081
Lee index **	0.30 ± 0.017	0.31 ± 0.005	0.29 ± 0.05	0.30 ± 0.005	0.146
Male (n: 3) ***					
BWG-8w	159.4 ± 13.45	243.2 ± 10.03	207.8 ± 31.74	218.4 ± 45.27	0.082
Lee index-8w	0.31 ± 0.010	0.30 ± 0.005	0.29 ± 0.005	0.31 ± 0.005	0.122
Female (n: 3) ***					
BWG-8w	146.3 ± 5.90	136.5 ± 3.91	176.0 ± 26.17 ^a^	209.7 ± 36.00 ^b^	0.026 *
Lee index-8w	0.29 ± 0.017	0.31 ± 0.005	0.29 ± 0.005	0.30 ± 0.005	0.120

BWG—Body weight gain (g), w—week * *p* < 0.05, ** One-Way ANOVA, *** Kruskal–Wallis Test, ^a^, ^b^, ^c^, ^d^ *p* < 0.05 for groups of different letters, *p* > 0.05 for groups of the same letters, CD—Control Diet, HFD—High-Fat Diet.

**Table 5:** Assessment of biochemical parameters in offspring rats based on week and maternal diet characteristics.

	CD-CD	CD-HFD	HFD-CD	HFD-HFD	*p*
	$\bar{x}$ ± SD (n: 6)	$\bar{x}$ ± SD (n: 6)	$\bar{x}$ ± SD (n: 6)	$\bar{x}$ ± SD (n: 6)	
3-week-old rats					
Glucose (mg/dL)	88.4 ± 5.83	99.7 ± 2.77	98.3 ± 9.65	103.9 ± 8.26	0.009 *
Insulin (mlU/L)	5.53 ± 0.50	5.88 ± 1.25	6.45 ± 1.42	5.63 ± 0.61	0.429
Triglyceride (mmol/L)	494.0 ± 26.24 ^a^	478.4 ± 25.39 ^b^	472.2 ± 20.01 ^b^	439.3 ± 27.31 ^c^	0.009 *
Total-cholesterol (mmol/L)	1.58 ± 0.08	1.67 ± 0.12	1.58 ± 0.21	1.60 ± 0.21	0.794
Leptin (ng/L)	1.98 ± 0.24	2.27 ± 0.18	2.20 ± 0.21	2.15 ± 0.18	0.126
8-week-old rats					
Glucose (mg/dL)	92.3 ± 5.31	94.9 ± 7.42	92.1 ± 4.39	93.5 ± 4.47	0.811
Insulin (mlU/L)	5.93 ± 0.49 ^b^	5.25 ± 0.62 ^a^	6.42± 1.01 ^c^	5.25 ± 0.37 ^c^	0.016 *
Triglyceride (mmol/L)	500.8 ± 25.88	487.5 ± 19.74	466.9 ± 29.38	469.8 ± 18.6	0.066
Total cholesterol (mmol/L)	1.53 ± 1.63	1.35 ± 1.88	1.47 ± 0.22	1.42 ± 1.48	0.368
Leptin (ng/L)	2.07 ± 0.12	1.68 ± 0.39	1.77 ± 0.27	1.82 ± 0.22	0.116

One-way ANOVA * *p* < 0.05, ^a^, ^b^, ^c^ *p* < 0.05 for groups of different letters, *p* > 0.05 for groups of the same letters CD—Control Diet, HFD—High-Fat Diet.

**Table 6:** The gene expression levels (fold change) of offspring rats based on maternal diet and gender.

Gene Expression	CD-CD $\bar{x}$ ± SD	CD-HFD $\bar{x}$ ± SD	HFD-CD $\bar{x}$ ± SD	HFD-HFD $\bar{x}$	*p*
3-week-old rats (n: 6)					
FAS	1.634 ± 2.226	0.898 ± 0.571	0.158 ± 0.121	0.547 ± 0.440	0.195
SREBP-1c	1.919 ± 2.597	0.530 ± 0.405	0.417 ± 0.352	1.884 ± 1.756	0.217
PPAR-γ	4.197 ± 5.295	0.584 ± 0.327	0.800 ± 0.940	1.028 ± 1.882	0.127
Male 3-week-old (n: 3)					
FAS	2.395 ± 3.255	0.921 ± 0.339	0.204 ± 0.166	0.730 ± 0.594	0.460
SREBP-1c	3.330 ± 3.284	0.758 ± 0.431	0.523 ± 0.378	2.403 ± 2.122	0.316
PPAR-γ	2.826 ± 4.720	0.646 ± 0.220	1.122 ± 1.363	1.682 ± 2.676	0.799
Female 3-week-old (n: 3)					
FAS	0.873 ± 0.244	0.876 ± 0.835	0.112 ± 0.052	0.364 ± 1.745	0.363
SREBP-1c	0.508 ± 0.324	0.304 ± 0.265	0.310 ± 0.365	1.365 ± 1.548	0.825
PPAR-γ	5.568 ± 6.495	0.523 ± 0.456	0.478 ± 0.208	0.373 ± 0.640	0.176
8-week-old rats (n: 6)					
FAS	2.063 ± 2.313	3.171 ± 6.437	2.073 ± 1.683	3.603 ± 6.477	0.922
SREBP-1c	1.909 ± 1.735	0.918 ± 0.364	2.265 ± 2.01	1.768 ± 3.175	0.718
PPAR-γ	1.878 ± 1.528	0.220 ± 0.197	3.409 ± 6.725	1.465 ± 2.390	0.520
Male 8-week-old (n: 3)					
FAS	2.426 ± 2.518	5.960 ± 8.958	1.809 ± 1.837	6.123 ± 9.142	0.164
SREBP-1c	2.357 ± 2.119	0.927 ± 0.524	1.313 ± 1.100	0.642 ± 0.521	0.393
PPAR-γ	1.496 ± 1.728	0.261 ± 0.260	0.628 ± 2.413	2.369 ± 3.437	0.220
Female 8-week-old (n: 3)					
FAS	1.701 ± 2.576	0.383 ± 0.132	2.338 ± 1.870	1.084 ± 1.509	0.591
SREBP-1c	1.462 ± 1.561	0.909 ± 0.236	3.217 ± 2.492	2.894 ± 2.492	0.695
PPAR-γ	2.261 ± 1.552	0.181 ± 0.157	6.191 ± 9.477	0.561 ± 0.157	0.448

One-way ANOVA Test, FAS—Fatty Acid Synthase, SREBP1-c—Sterol Regulatory Element-Binding Protein 1-c, PPAR-γ—Peroxisome Proliferator-Activated Receptor gamma, CD—Control Diet, HFD—High-Fat Diet.

**Table 7:** Criteria for Adipose Tissue Degeneration in Offspring Rats According to Maternal Diet Characteristics.

	3 Weeks Old				8 Weeks Old			
	CD-CD (n: 6)	CD-HFD (n: 6)	HFD-CD (n: 6)	HFD-HFD (n: 6)	CD-CD (n: 6)	CD-HFD (n: 6)	HFD-CD (n: 6)	HFD-HFD (n: 6)
Deformation of adipocytes		++	+	++		+++	+	+++
Fusion of adipocytes		+		++		++	+	+++
Lipid disturbance and myelin formation in adipocytes		++	+	++		++	+	+++
Density of brown adipose tissue cells			+				+	
Increase in connective tissue			++	++			+++	++
Dilation in blood vessels		+	++	+		++	++	+++
Infiltration			++	++			++	++

CD—Control Diet, HFD—High-Fat Diet. +: Mild, ++: Moderate, +++: Severe.

## Data Availability

The data presented in this study are available on request from the corresponding author: Sabriye Arslan, Department of Nutrition and Dietetics, Gazi University Faculty of Health Sciences, Ankara, Turkey; sbolluk@gazi.edu.tr.
